# Self-powered smart patch for sweat conductivity monitoring

**DOI:** 10.1038/s41378-018-0043-0

**Published:** 2019-01-28

**Authors:** Laura Ortega, Anna Llorella, Juan Pablo Esquivel, Neus Sabaté

**Affiliations:** 1 Instituto de Microelectrónica de Barcelona, IMB-CNM (CSIC), C/del Til·lers. Campus Universitat Autònoma de Barcelona (UAB), 08193 Bellaterra, Barcelona Spain; 2 Catalan Institution for Research and Advanced Studies (ICREA), Passeig Lluís Companys 23, 08010 Barcelona, Spain

**Keywords:** Engineering, Chemistry, Physics

## Abstract

A self-powered skin patch for the measurement of sweat conductivity is presented. The key component of the patch consists of a paper battery that is activated upon absorption of sweat. This body fluid acts as the battery electrolyte, the conductivity of which has a direct impact on the battery-generated output power and voltage. This particular behaviour enables the operation of a very simple and robust conductivity sensor in direct current mode without needing an external power source. The device presented in this paper takes advantage of this new measurement method to develop a sweat patch for screening cystic fibrosis that operates with an extremely simple electronic circuit that minimizes its cost and environmental impact. The patch provides an unambiguous digital result that can be read in an electrochromic display and yields 95% sensitivity and 100% specificity when tested with artificial eccrine perspiration samples.

## Bioelectronics: Conductivity powered by sweat

The conductivity of body sweat can be measured by sweat itself acting as the battery electrolyte. Wearable electronic devices for health monitoring are gathering pace for their ability to detect and interpret body signals. Amongst these, it has previously been shown that measuring the conductivity of sweat might be used to indicate cystic fibrosis. However, previous devices required an external power supply. Now, a team from Instituto de Microelectrónica de Barcelona, Spain, led by ICREA Professor Neus Sabaté demonstrates a self-powered sweat conductivity-measuring device. The key innovation in making the device self-powered is the use of a paper-based battery which absorbs the sweat, which acts as the electrolyte. This merging together of the power generating and sensing mechanism in a single battery gives rise to a very simple yet smart device with digital output.

## Introduction

Wearable devices for health condition monitoring have attracted considerable interest in the sports and healthcare sectors in recent years. These devices provide real-time monitoring of physiological relevant parameters of human well-being. Significant advances in wearable solutions that measure physical parameters, such as heart rate, bodily motion, skin temperature or respiration rate, have already been achieved; many of these devices are available in the market^1–3^. However, obtaining chemical parameters from body fluids provides insight into a wearer’s health, performance or stress at the molecular level^4^. Sweat collection and analysis offers many ergonomic advantages over other substances, such as the availability of sampling sites and their continuous access and the ability to be stimulated on-demand with local iontophoresis^5^. These advantages have motivated researchers to develop a first generation of interesting prototypes that are primarily based on colorimetric detection^6,7^, in which signal quantification requires digital capture and analysis with a dedicated reader or a smartphone. Due to its irreversible nature, these devices are designed to function for a short period and be disposed afterwards. More endurable and quantitative approaches that are based on their detection principle for electrochemical sensors have addressed the integration of electrochemically active electrodes on flexible and skin-friendly skin patches. However, only a few studies assess onsite signal processing circuitry and sensor calibration mechanisms for an accurate analysis^8–11^. Recently, this device has been applied to cystic fibrosis monitoring, in which the wearable platform enables both sweat stimulation and chloride ions detection^12^. However, its complexity and the number of electronic components that are required to drive the sensors and extract the appropriate information renders it more suitable for long-term clinical and physiological investigations than for testing cystic fibrosis disease on individuals. Most of the approaches to chemical sensing require complex electronic circuits with power-on board, which yields bulky and costly devices. The search for more compact and sustainable power sources has prompted the development of self-powered devices, in which energy to perform a measurement is harvested from the environment or the biological system under study. Numerous prototypes have been developed to measure biological parameters by harvesting power from different sources, such as breathing, heartbeat, temperature or biological fluids^13–16^.

In this paper, we present a self-powered skin patch to measure sweat conductivity and its application to screening cystic fibrosis with a novel and simple approach. Cystic fibrosis (CF) is a genetic autosomal recessive disease that induces mutations on a conductance transmembrane regulator protein—cystic fibrosis conductance transmembrane regulator (CFTR)—which controls the excretion of chloride in sweat. For this reason, patients with CF present higher chloride contents in sweat than healthy subjects. Although most of the approaches focus on measuring chloride concentration, different studies have demonstrated the effectiveness of the sweat conductivity measurement as a screening test for CF^17,18^. Conductivity measurements currently require AC electronics modules that render its integration on a disposable patch impractical. In this paper, we show how conductivity measurements of sweat can be approached in a simpler way.

The key component of the patch consists of a paper battery that is activated upon adding the fluid to be measured (Movie S1, Supporting Information). The fluid acts as an electrolyte, and for a particular configuration of the battery materials and design, the battery output is fully dependent on the conductivity of the liquid sample that is poured in its paper core. This approach enables the sensor and battery to be merged in a single element—a battery sensor—whose generated power is directly related to the conductivity of the sample to be analysed. Compared with state-of-the-art conductivity sensors, the battery sensor presents two main advantages: (1) its response to conductivity can be characterized by a direct current (DC) that enables a radical simplification of the electronic module to operate it, and (2) if desired, the sensor response can be obtained without an additional power source; thus, it can be operated as a self-powered sensor. This new approach to conductivity sensing breaks the paradigm sensor-electronics-battery and converts it into something simpler as the sensor and battery are merged into the same element. This paper shows how this new measuring concept can be successfully employed to construct an autonomous screening patch that can deliver a digital output using a minimal number of discrete electronic components that can be completely printable.

**Fig. 1:**
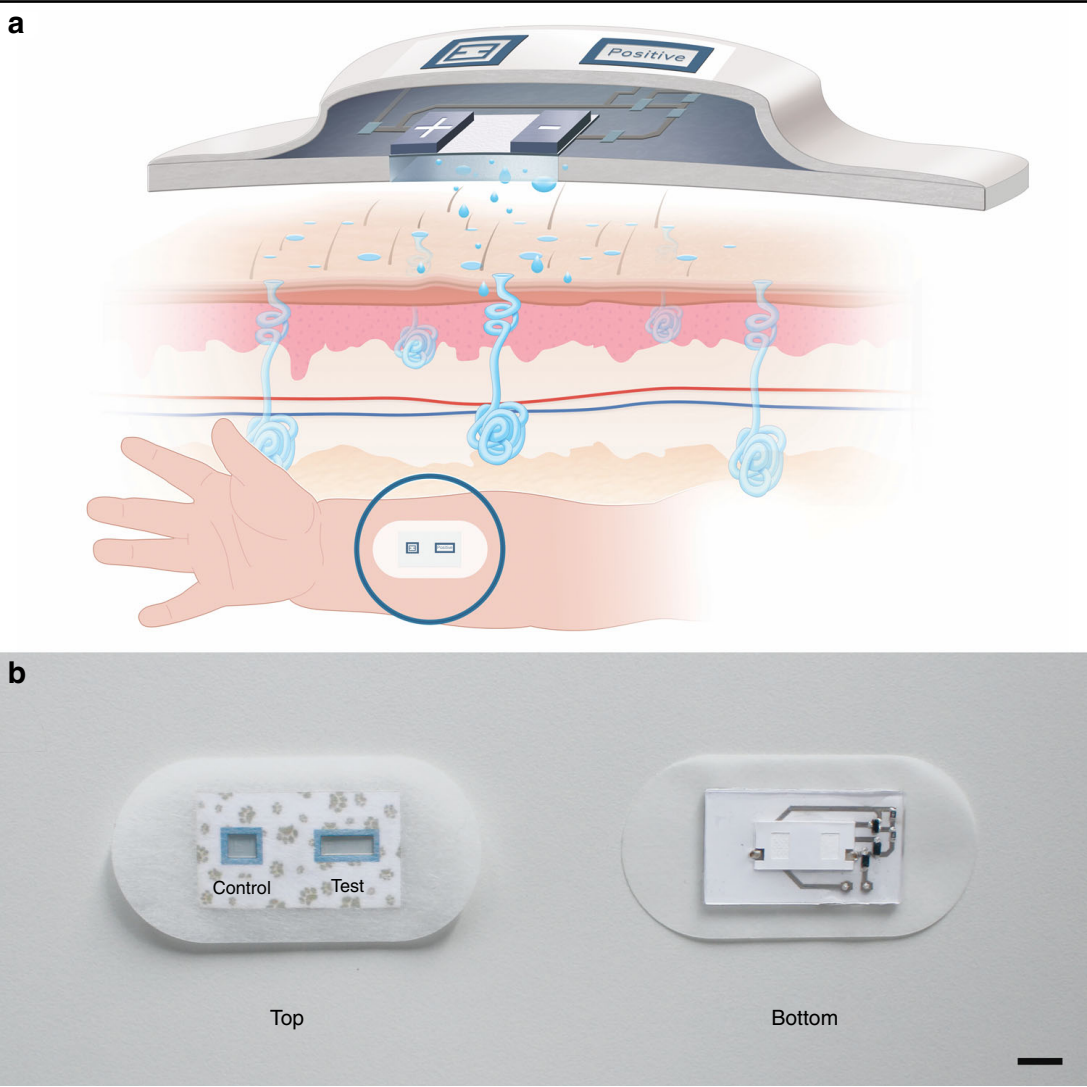
Device design and implementation. **a** Scheme of the designed patch and theoretical working scenario. **b** Final implemented device. The Control and Test display are shown in the top face, and the battery stack and inkjet printed circuit with the discrete components are observed in the bottom face (scale bar = 1 cm)

The patch has been conceived for application on the forearm of a patient and yields a “positive” result in the case in which an abnormal conductivity level of sweat is detected (Fig. 1). The patch remains quiescent until the sweat is absorbed by the paper that is in contact with the skin. The generated power is handled by a very simple circuit that enables discerning between a healthy condition and a non-healthy condition. A flow chart of the device operation is shown in Figure S1. This circuit combines inkjet printed conductive tracks, discrete electronic components and two electrochromic displays (Control and Test output) to show the result.

## Results

### Conductivity sensor description and working principle

**Fig. 2:**
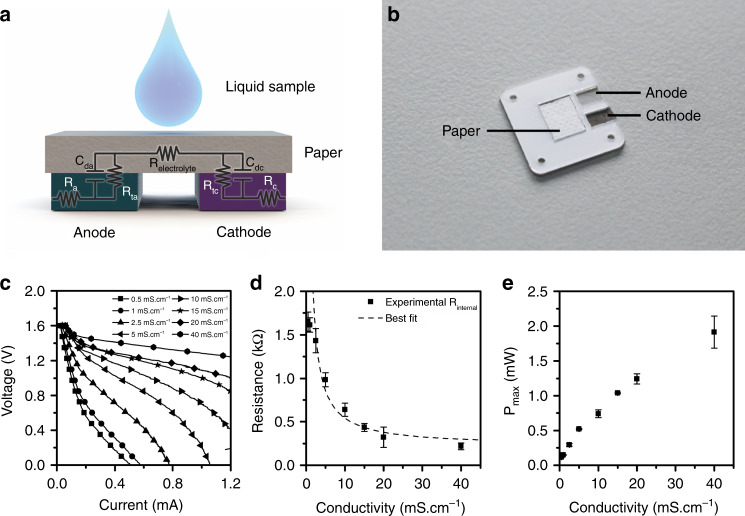
Basic concepts of the battery. **a** Battery model. **b** Image of a single battery. **c** Polarisation curves of a single battery with different conductivity of electrolytes. **d** Resistance of the battery in the ohmic region of the polarization curve as a function of the conductivity of the electrolyte. **e** Maximum power output of the battery for different electrolyte conductivities

Liquid-activated batteries are devices that consist of at least two electroactive electrodes: an oxidizing electrode (anode) and a reducing electrode (cathode). The electrodes are connected by a hydrophilic material that is capable of holding fluid (Fig. 2a). This kind of battery starts to function upon the addition of fluid, which acts as the battery electrolyte. Liquid used to activate the battery operation is generally a water-based fluid, such as plain water, beverages or any biological fluid (such as saliva, urine, blood and sweat). These batteries are primary batteries that cease to function when their electrodes are exhausted.

The voltage delivered by a battery depends on the thermodynamic voltage of the electrochemical reactions involved in its electrodes. However, once the battery starts to deliver power, its voltage-intensity characteristic curve will depend on the internal resistance of the battery. This parameter depends on the efficiency of the charge generation processes that occur at the battery electrodes and the charge transport across the electrolyte and the resistive elements of the battery.

Charge transport inside the battery occurs through different media; charge carriers in the metallic components are electrons, whereas current in the electrolyte consists of ion transport. Despite its different nature, both charge transport phenomena can be modelled by resistors (Fig. 2a). R_a_ and R_c_ account for the ohmic resistivity of the anode and cathode materials, whereas R_electrolyte_ represents the ionic resistivity of the electrolyte. The charge transfer between the ohmic conductors and the electrolyte occurs by the oxidation and reduction reactions at the electrodes. These processes can be modelled with a parallel combination of a resistor (R_t_) and a capacitor (C_d_), where R_t_ accounts for the faradaic resistance, which represents the kinetics of the electrochemical reactions, and C_d_ accounts for the double-layer capacitance, which reflects the capacitive nature of the interface between the electrode and the electrolyte. Contribution of the faradic resistance R_t_ to the battery internal resistance is relevant at low currents and becomes practically negligible at moderate and high currents of the cell, which enables the ohmic region of the battery I–V curve, where the total internal resistance can be attributed to the resistivity of the electrodes and the electrolyte, Equation (1). For a fixed configuration of the battery materials and geometry, changes in the internal resistance of the battery at the ohmic region of the polarization curve can be solely attributed to changes in the conductivity of the electrolyte. The resistance of the electrolyte depends on the geometry and porosity of the hydrophilic core, the area and position of the electrodes and the conductivity of the liquid used to activate the battery, which will be inversely proportional to the resistance of the cell.

1 $$R_{internal} = R_a + R_c + R_{electrolyte} = A + \frac{B}{\sigma }$$

The battery developed in this study consists of two coplanar electrodes with the dimensions 2.5 × 5 mm^2^. Each electrodes were constructed of magnesium (anode) and silver chloride (cathode) mounted on top of a pressure sensitive adhesive layer and separated by 1.5 mm (Fig. 2b). Magnesium electrodes are cut from a 100-µm-thick foil, whereas silver chloride electrodes are obtained by a controlled chlorination of a screen-printed silver track. This process has allowed us to obtain highly reproducible battery cathodes. The electrodes—which are placed side by side—and the area between them are covered with two layers of glass fibre-based paper with a total thickness of 0.5 mm and an area of 60 mm^2^. The glass fibre paper is a very suitable material due to its high water absorption rate and its high porosity, which enables holding a liquid volume of 15 µL. When the liquid sample to be characterized is deposited on the paper, it is absorbed by capillarity until the paper is completely saturated. Unlike other candidates, such as cellulose, this material does not experience significant swelling after water is absorbed, which enables keeping the geometry of the electrolyte constant.

Battery operation starts once the paper is completely filled with the sample. The basic electrochemistry of this battery has been previously employed in seawater-activated batteries^19,20^. The reactions involved in the paper battery are expressed as follows:

$$Anode:Mg\left( s \right) \to Mg^{2 + }(aq) + 2e^ -$$

$$Cathode:AgCl\left( s \right) + e^ - \to Ag\left( s \right) + Cl^ - (aq)$$

$$Total:\, Mg\left( s \right) + 2AgCl\left( s \right) \to Mg^{2 + }\left( {aq} \right) \\ + 2Cl^ - \left( {aq} \right) + 2Ag(s)$$

The standard voltage for this cell is 2.59 V. However, the open-circuit voltage (V_OC_) in neutral media is set from 1.5–1.7 V. This departure from the theoretical voltage is attributed to the high polarisation voltage caused by the build-up of an oxide layer on the Mg film. To test the impact of the ionic conductivity on the performance of our battery, we recorded the I–V curves when filled with water-based solutions that contain different NaCl concentrations that establish liquid conductivities from 0.5 mS cm^−1^ to 40 mS cm^−1^ (Fig. 2c). The polarization curves show a visible influence of ionic conductivity of the liquid sample that is used as an electrolyte on the resistivity of the cell. This finding is observed in the ohmic region of the curves (currents above 0.2 mA). At low current values (near OCP voltage), large activation losses related to the pre-existing passivating layer of magnesium oxide in the anode can be observed. The effect of this passivating layer becomes less prominent at increasing conductivities as the presence of chloride ions increases the rate of anodic dissolution kinetics^21^. At higher current densities, the battery shows concentration losses due to a limitation in the AgCl reduction reaction rate at the cathode. Internal resistance of the battery (obtained from the slope of I–V curves at the ohmic region) versus the ionic conductivity of the electrolyte has been computed. The results indicate that the experimental data follow the expected σ^−1^ dependence within the conductivity range between 2.5 and 40 mS cm^−1^ (Fig. 2d). At conductivities below 2.5 mS cm^−1^, the battery performance is dominated by large activation losses, and therefore, the internal resistance dependence on conductivity departs from ideal behaviour.

Although the influence of the liquid conductivity on the internal resistance of the battery has been visualized by its impact on the polarization curves, the change in battery performance can be obtained using a large variety of well-known electrochemical characterization techniques (such as chronoamperometry, chronopotentiometry, electrochemical impedance spectroscopy, current-interrupt measurements). However, one of the most interesting features of the presented battery is that the power it delivers is proportional to the conductivity of the liquid sample, and therefore, it can be operated as a self-powered sensor (Fig. 2e). This last feature makes the battery particularly suitable to sense conductivity in portable and disposable applications, as it removes the requirement of an additional power source.

### Self-powered patch design rational and circuit validation

The sweat patch has to operate within a conductivity range from 5 mM equiv NaCl to 160 mM equiv NaCl (equiv NaCl), as these limits are limits of interest and the measurement units as defined in the Diagnostic Sweat Testing Guidelines from the Cystic Fibrosis Foundation^22,23^.

Body sweat is a complex mixture of cumulative secretions from eccrine, apocrine and sebaceous glands, which contain various electrolytes, ions, amino acids, proteins and lipids. Although the composition of sweat depends on many factors, such as the sweat production rate and the physiological state, the lipid content of sweat generated on the forearm—the area selected for cystic fibrosis screening—is present in concentrations below 1%. With the exception of ionic species, most of the components present in sweat are neutrally charged and have a negligible effect on conductivity. The ionic content varies from patient to patient. In the past 20 years, different series of clinical studies aimed to determine the interferences of different species aside from Cl^−^ and Na^+^ in sweat conductivity have been performed^17,18,24,25^. The studies analysed the ionic content of hundreds of patients and concluded that the conductivity of sweat approximates very closely to the sum of its sodium and chloride ions with a tolerance margin that accounts for conductivity variations caused by the presence of secondary ions (Table S1). The patch has been designed to operate as a screening device that generates a positive result with sweat conductivities that exceed 60 mM equiv NaCl. For result visualization, electrochromic displays were identified as the most suitable technology due to their compatibility with flexible substrates, their fabrication with screen-printing techniques and their capability to operate with voltages as low as 0.6 V.

**Fig. 3:**
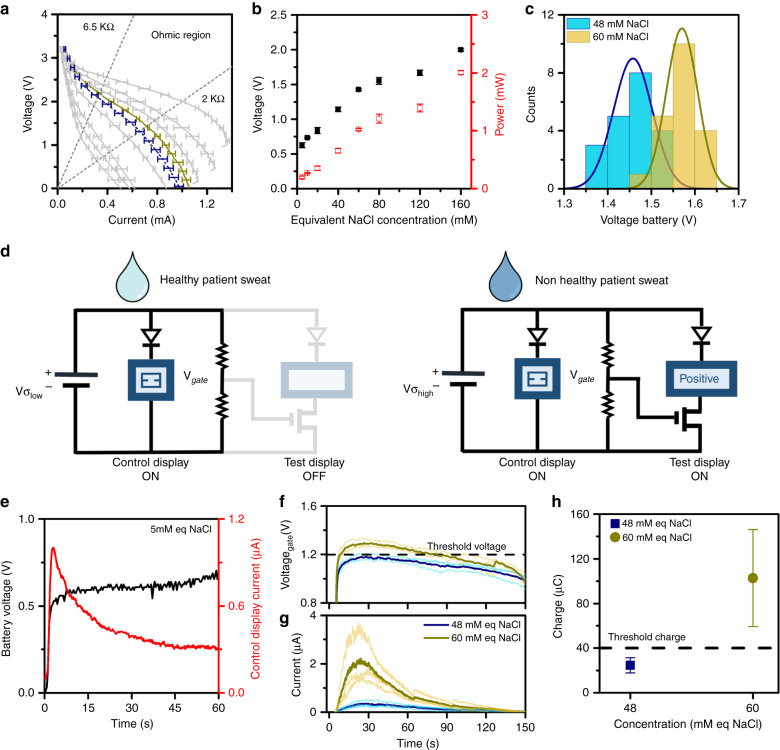
Operating principle of the self-powered patch. **a** Battery stack calibration with samples at increasing concentrations of 5 mM, 10 mM, 20 mM, 40 mM, 48 mM (in dark blue), 60 mM (in dark yellow), 80 mM, 120 mM and 160 mM NaCl. The battery stack calibration showed that R_LOAD_ ranges from 2 kΩ to 6.5 kΩ, which enabled setting the battery operation within its ohmic region. **b** Voltage and power generated by the battery when introducing an R_LOAD_ of 2 kΩ. **c** Gaussian distribution around central values of 1.46 V and 1.57 V, which corresponds to 48 mM equiv NaCl and 60 mM equiv NaCl, respectively, when the battery was submitted to an R_LOAD_ of 2 kΩ. **d** Circuit operation when healthy and non-healthy sweat samples are analysed. **e** Voltage and current provided by the battery and collected in the Control Display, respectively, at the lowest conductive sample. **f**, **g** Operation of the whole device with samples at 48 mM and 60 mM equiv NaCl in voltage at the gate terminal of the transistor and the current stored in the Test Display. **h** Charge accumulated in the Test Display at the two concentrations of NaCl

Based on simplicity, the operating principle of the battery consisted of connecting the purely resistive load R_LOAD_ (Fig. 3a). If the selected R_LOAD_ enables the battery to operate inside the ohmic region of its polarization curve, the voltage generated by the battery is directly dependent on the sweat conductivity that is used to activate it. Based on this requirement and the need to ensure a minimum output voltage of 0.6 V, which is required to turn on the Control Display at the lowest conductivity range (5 mM), the patch required the stacking of two battery units. The value of R_LOAD_ was fixed to the lowest value of 2 kΩ, which enables the battery to be operated at its maximum output power. The generated power ranges from 0.2 mW to 2 mW within the conductivity range of interest (Fig. 3b).

The patch performance was designed to follow the *User protocol for Evaluation of Qualitative Test Performance (EP12-A2)* dictated by the Clinical and Laboratory Standards Institute^26^. The protocol recommends evaluating the compliance of the screening test by statistically testing its outcome at the threshold value (60 mM) and at a cut-off value that corresponds to the −20% of the threshold value (48 mM in this case).

The polarisation curves of a certain number of statistically relevant battery stacks (*N* = 40) activated with deionized water samples with NaCl concentrations of the threshold and cut-off values were recorded (Fig. 3c). Although the populations appear separated, an overlapping of the voltage output of the battery was observed.

Since the objective of the patch is to perform a screening measurement, a decision strategy was needed to set a suitable voltage threshold that would yield a positive result in the Test Display. To priorities the minimization of false negative results, the threshold voltage was placed at −5% of the Gaussian distribution of 60 mM equiv NaCl (1.51 V), which ensures that 95% of ill patients yield positive results. Due to the overlapping of the populations, this decision would yield 12% false positive results of healthy patients.

Based on this decision, the logic of the device was designed following the criteria of a minimum number of electronic components (Fig. 3d), which would minimize both cost and environmental impact after disposal. (Fig. S2, Supporting Information). The Control Display is directly connected to the battery stack and it ensures that the patch is working properly regardless of the conductivity of the sweat sample. The Test Display is activated when the battery yields a voltage that is equal to or above the CF-positive threshold voltage. Two diodes are connected to the displays to prevent their discharge once the battery ceases to operate. The Test Display activation is controlled with an n-type MOSFET transistor that enables current to flow when the voltage applied to its gate (V_gate_) is higher than 1.2 V (Fig. S3, Supporting Information). To match the CF-positive threshold voltage of the battery stack with V_gate_, R_LOAD_ was split between resistor R_1_ and resistor R_2_.

The accumulated threshold charge value that provided a distinguishable display activation at a minimum working potential of 0.6 V was set to 10 µC and 40 µC for the Control display and Test display, respectively.

The circuit was tested on a protoboard and validated with samples with relevant conductivities of 5 mM—minimum conductivity value, 48 mM and 60 mM (all in equiv NaCl). The activation of the Control Display at 5 mM equiv NaCl was checked. At this concentration, the battery yielded a voltage of 0.6 V during the first 60 s (Fig. 3e), which produced an accumulated charge of 50 μC that activated the Control Display. The patch performance was tested with triplicated samples of 48 mM equiv NaCl and 60 mM equiv NaCl, respectively. The evolution of the voltage at the gate terminal of the MOSFET transistor and the current that flows towards the Test Display was monitored during the tests (Fig. 3f, g). A steady decline in the voltage delivered by the batteries under continuous operation is observed. This decline is attributed to an increase in the internal resistance caused by the consumption of the battery electrodes during device operation (Fig. 3f). However, this behaviour has proven to be highly reproducible with a variation coefficient less than 3.5%. Once the battery is saturated with a conductive liquid solution, the voltage applied to the transistor increases to a value that depends on the conductivity of the sample. For 60 mM equiv NaCl, this voltage is higher than the transistor threshold voltage (1.2 V, represented with a dashed line), which causes current to flow towards the Test Display. The voltage is sustained above the transistor threshold for ~ 60 s but subsequently decays due to the progressive exhaustion of the battery electrodes. At 48 mM equiv NaCl, the voltage at the transistor gate never attains the threshold voltage value; thus, the Test Display remains switched off. The current that flows towards the electrochromic display during the system operation in both cases has been depicted (Fig. 2g). A significant current flows towards the Test Display for all tested samples at 60 mM. Although no current is expected to flow towards the display at 48 mM, a small current was observed. This current corresponds to a leakage current of the transistor (Fig. S3, Supporting Information) at voltages between 1.15 V and 1.2 V. This current was unable to switch on the Test Display as the accumulated charge did not exceed the threshold charge to generate any significant change in the active segments contrast (Fig. 3h).

### Self-powered patch fabrication and testing

**Fig. 4:**
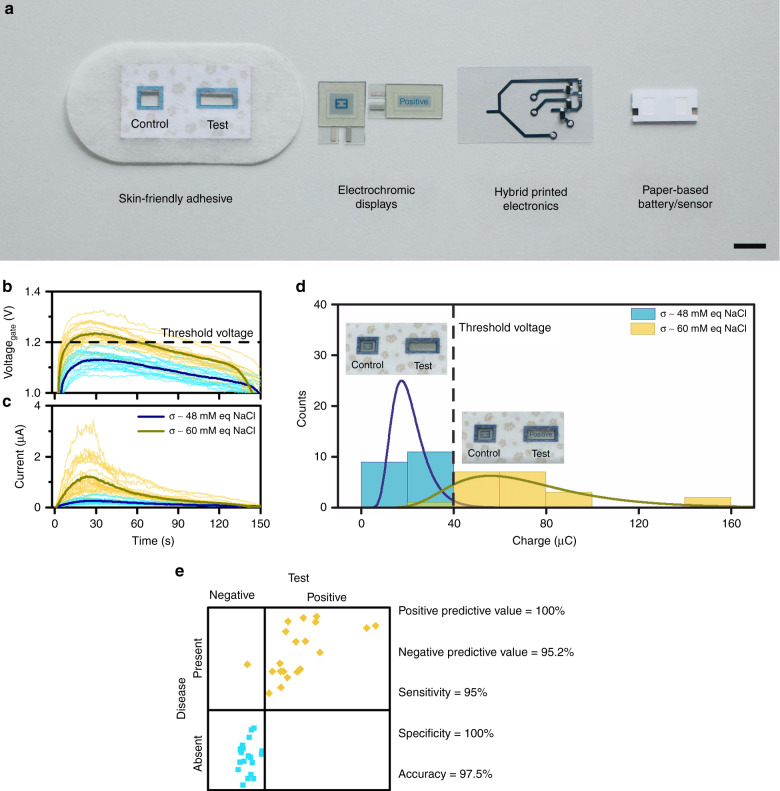
Final device components and validation. **a** Exploded view of the final device with the different layers (scale bar = 1 cm). **b**, **c** Operation of the device with artificial sweat samples at the two conductivities of 48 mM equiv NaCl and 60 mM equiv NaCl. **d** Gaussian distribution of the accumulated charge at the two sample conductivities. **e** Final representation of the results provided by all devices compared with the known condition of the samples—healthy and unhealthy

The patch is conceived to be single use and completely disposable. All components were integrated in a compact self-contained skin patch (Fig. 4a). The patch incorporates the paper battery stack that absorbs 30 µL of the fluid sample. Battery electrodes are placed at the inner face of the sampling paper. In this configuration, the sample is forced to pass through the entire thickness of the paper core before reaching the electrode surface and activate the chemical reactions. This process mitigates the risk of a partial soaking of the paper battery core that would yield an incorrect conductivity value. An electronic circuit design was inkjet printed using Ag ink on a flexible polyethylene naphthalate (PEN) sheet. Silver conducting paste was used for the hybridization of SMD electronic components and the battery terminals. The area surrounding the battery was encapsulated with PDMS elastomer to protect the electronic circuit and avoid any short circuit caused by occasional sweat overflooding. The elastomer also provides a flat surface that improves skin contact and avoids sweat evaporation. Two custom-made electrochromic displays (Ynvisible, Vancouver, Canada) were glued at the rear of the flexible substrate of the circuit and were connected to the corresponding circuit terminals by a bias filled with conducting paste. A layer of a medical-grade pressure-sensitive adhesive that was coated on a stretchable nonwoven fabric was laser-cut to assemble all components in an adhesive bandage form factor and provide sealing conditions during sweat collection and measurement.

This top side includes two windows for display visualization. Graphic features were printed on the top face of the adhesive to make it pleasant for small children. All components were manually assembled using an alignment jig.

The final device was validated with artificial eccrine perspiration, which mimics human eccrine sweat and consists of 19 amino acids—the most abundant minerals and metabolites at concentrations that match experimentally determined values for adult human eccrine sweat (Table S2). The purchased artificial sweat presented a conductivity of 80 mM equivalent of NaCl. Thus, the conductivity for each sample was adjusted to 48 mM equiv NaCl and 60 mM equiv NaCl by diluting the samples with deionized water.

To be compliant with the EP12-A2  test protocol, the validation of the devices was performed with 40 different devices, with which 20 samples at conductivities of 48 and 60 mM equiv NaCl were employed. The voltage was recorded at the gate terminal of the MOSFET transistor (Fig. 4b). Samples that represent “healthy” patients yielded voltages under the transistor threshold voltage in all tested devices. Conversely, samples at higher “non-healthy” conductivity values yielded voltages above the threshold, which enabled the current to flow towards the Test Display. The current that flows towards this display in all tested cases was translated to the accumulated charge in the Test Display (Fig. 4c, d). All healthy samples generated charges less than 40 μC, and therefore, did not turn on the display. The accumulated charges obtained when testing the devices with the threshold conductivity value of 60 mM ranged from 40 μC to 150 μC. The high dispersion in the recorded charge values is attributed to an existing exponential relationship between the voltage gate and the current of the transistor in the working region selected in this application (region near the transistor cut-off value) (Fig. S3, Supporting Information). The contingency table to compare the results of the test with the known diagnosis shows that our device yields 95% sensitivity and 100% specificity (Fig. 4e).

## Discussion

The difference in performance of the different patches has been assessed in the paper by testing the variability of the fabricated batteries with NaCl solutions (Fig. 3c), testing the response of the batteries after connecting the minimal electronic circuit (Fig. 3h) and checking the performance of 40 ready-to-use patches with artificial sweat samples (Fig. 4d). The coefficients of variation obtained by characterizing the single battery at the different targeted concentrations are less than 5%. However, when the battery is connected to the electronics circuit mounted in a protoboard, the coefficients of variation of the response increases to 25% in the case of a low conductivity value (48 mM) and 40% in the case of the upper threshold value (60 mM). This finding is attributed to the fact that the system relies on a single MOSFET transistor to determine whether the voltage generated by the battery corresponds to a positive response or a negative response. When the battery operates at voltages near the value of the threshold voltage of the transistor, the transistor operates in a transition region that limits the current generated by the battery. This source is the main source of the variation recorded in the total current that flows towards the display elements. When 40 patches are tested with artificial sweat samples that contain amino acids, urea and different ions, the coefficients of variation slightly increase to 30% (at 48 mM) and 42% (at 60 mM), which indicates that the effect of introducing a more complex matrix that mimics real sweat does not introduce additional dispersion to the results.

The device presented in this paper shows the feasibility of a screening patch for the diagnostics of cystic fibrosis with a novel method of conductivity measurement. The use of a paper battery as a conductivity sensor enables a clinically relevant parameter to be obtained with an extremely simple electronic circuit that utilizes a small number of components. Considering state-of-the-art printing technologies, the elements of the circuit can be entirely printed with inkjet printing processes. Combined with the screen-printing methods that are employed to obtain the battery elements and the electrochromic displays, these processes pave the way to mass production of the device. In fact, these factors render the patch suitable for a single use and disposable approach, as the low cost associated with the materials and fabrication processes and the use of a minimum number of electronic components contributes to the sustainability of the device. Additionally, in an alternative scenario, the battery sensor can serve as a self-powered consumable component that can provide power and sensing capabilities for a batteryless reusable electronic reader. This approach has been successfully demonstrated for glucose monitoring^27^.

Note that the dual use of the battery as a sensor and a power source renders the system energetically independent, which is a main hurdle of a wearable device. The minimum power generated by the patch is 1 mW, which is dissipated almost entirely in the resistive elements in the current circuit design. In a more sophisticated version, this power can be used to enable communication or data storage functions. Despite the inherent impact of the current development in the clinical sector, the conductivity patch can be potentially utilized to evaluate the electrolyte content in sweat for other purposes, such as dehydration that is induced by physical activity.

## Materials and methods

### Battery fabrication

The battery was designed with CorelDraw (Corel, Ottawa, ON, Canada) and fabricated by rapid prototyping, such as laser cutting (Mini 24, Epilog Laser, Golden, CO, USA). The device was mounted with pressure-sensitive adhesives of medical grade (Adhesive Research, Glen Rock, PA, USA) by stacking all layers. The paper used to absorb the sample was a LF1 Glass Fibre Filter from GE (General Electric, Boston, MA, USA). For the electrodes, the Mg anode was obtained from GalliumSource LLC (Scotts Valley, CA, USA). The AgCl cathode was obtained by chlorinating an Ag electrode screen-printed over a PET surface (DuPont, Wilmington, DE, USA). The Ag electrode was screen-printed with a manual screen-printer (PAYMSER, Barcelona, Spain), the silver ink LOCTITE ECI 1011 E&C (Henkel, Düsseldorf, Germany) and cured at 150 °C for 15 min. The chlorination of the Ag electrodes was performed by introducing a constant current of 200 μA for 260 s with a Gamry Potentiostat Reference 3000 AE (Gamry, Warminster, PA, USA) in a stirred solution of 0.1 M NaCl (Sigma-Aldrich, St Louis, MO, USA) with a half-cell configuration (Ag/AgCl reference electrode and Pt counter electrode (Metrohm AG, Ionenstrasse, Herisau, Switzerland).

### Hybrid circuit fabrication

The circuit was simulated with LTSpice (Analog Devices, Norwood, MA, USA), designed with Kicad (Canonical Ltd, London, UK) and printed with the inkjet printer CeraPrinter X-Serie (Ceradrop, Parc d’Ester Technopole, Limoges, France). The ink used for the inkjet printing was a silver ink from DuPont PE-410 (DuPont, Wilmington, DE, USA), which was cured at 130 °C for 20 min. The discrete components and electrochromic displays were attached to the printed circuit with a silver epoxy EPO-TEK H20E (Agar Scientific Ltd, Essex, UK) by curing it at 80 °C for 3 h.

### Electrochemical characterization

The electrochemical characterization of the battery was performed with a Gamry Potentiostat Reference 3000 AE (Gamry, Warminster, PA, USA). The open-circuit voltage of the batteries was measured for 20 s, and a linear sweep voltammetry at 10 mV/s was performed from the open-circuit voltage to 0 V. Samples of NaCl (Sigma-Aldrich, St Louis, MO, USA) were prepared every day, and the conductivity and temperature of the sample was measured with the 914 pH/Conductometer (Metrohm AG, Ionenstrasse, Herisau, Switzerland).

### Electrical characterization

The electrical characterization of the system was performed with a 2400 SourceMeter (Keithley Instruments, Cleveland, OH, USA) and an HP34401A Multimeter (Hewlett-Packard Palo Alto, CA, USA) to measure the current stored in the Test display and the voltage provided by the battery, respectively, controlled with a custom LabVIEW program (National Instruments, N Mopac Expy, Austin, TX, USA).

### Artificial samples preparation

Artificial Eccrine Perspiration (Pickering Laboratories, Mountain View, CA, USA) was used to perform final validation of the device. The conductivity of the sample was adjusted to the equivalent conductivity of the healthy and unhealthy subjects by measuring the conductivity with the 914 pH/Conductometer (Metrohm AG, Ionenstrasse, Herisau, Switzerland) and adding deionized water.

## Supplementary information

Video S1

Supplemental Material
