# Supplementary material for: Self-powered smart patch for sweat conductivity monitoring
Source: Microsyst Nanoeng. 2019 Jan 28;5:3. doi: 10.1038/s41378-018-0043-0 (PMC6348283; doi:10.1038/s41378-018-0043-0)
Supplement: Supplementary file 2 — Supplemental Material [file 41378_2018_43_MOESM2_ESM.docx]

Supporting Information

Self-powered smart patch for sweat conductivity monitoring

Laura Ortega^*^, Anna Llorella, Juan Pablo Esquivel and Neus Sabaté

L. Ortega, A. Llorella, Dr. J. P. Esquivel, Prof. N. Sabaté

Instituto de Microelectrónica de Barcelona, IMB-CNM (CSIC)

C/ del Til·lers. Campus Universitat Autònoma de Barcelona (UAB)
08193 Bellaterra, Barcelona, SPAIN

E-mail: neus.sabate@imb-cnm.csic.es

Prof. N. Sabaté

Catalan Institution for Research and Advanced Studies (ICREA)

Passeig Lluís Companys 23, 08010 Barcelona, Spain

**Self-powered smart patch operation**


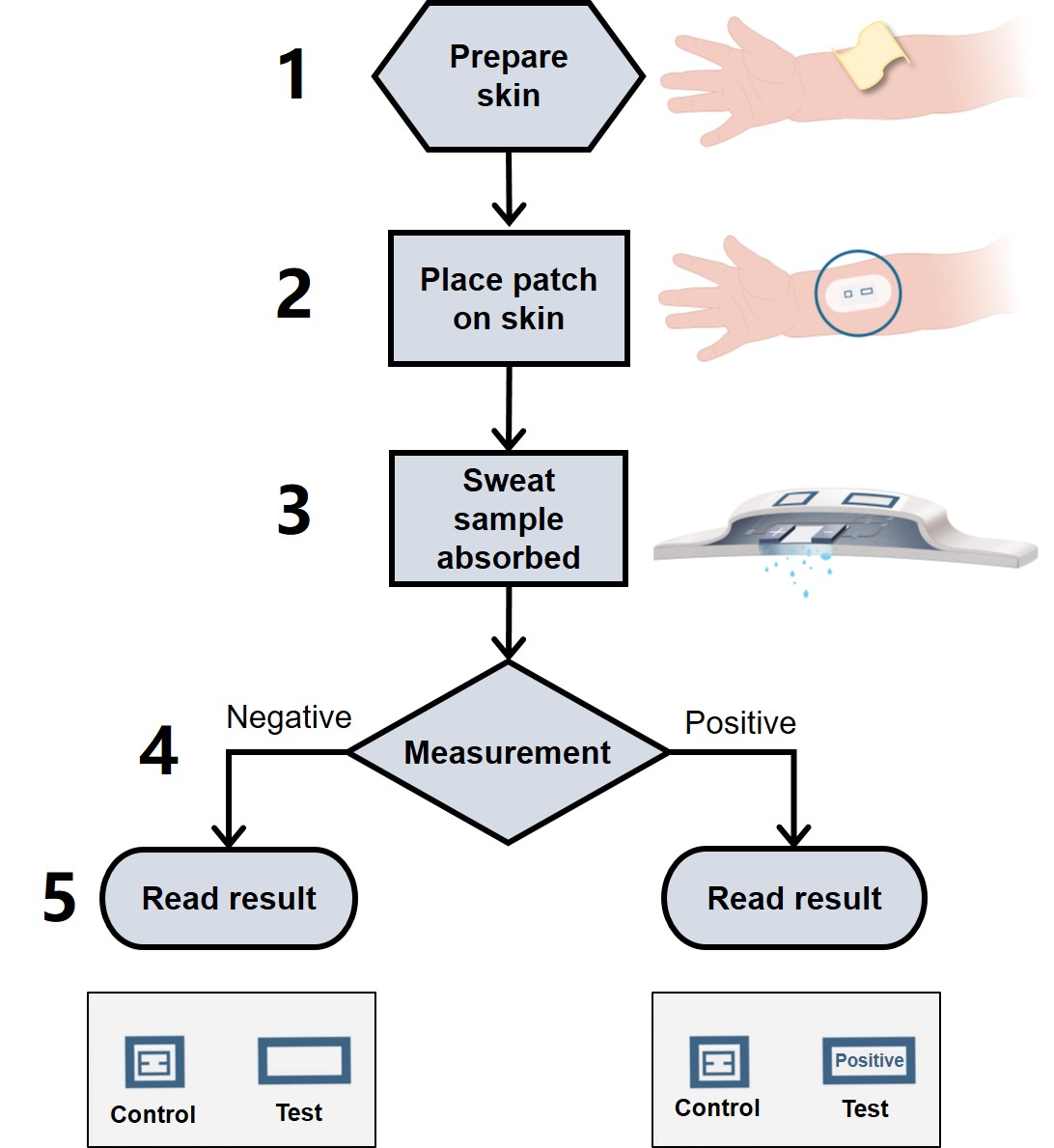


**Figure S1.** Flowchart of self-powered smart patch operation.

In the first step, the forearm skin should be prepared in a similar way as typical sweat measuring tests, including cleaning and sweat stimulation by iontophoresis (Step 1). Then, the patch is placed over the prepared skin surface using the dedicated adhesive layer (Step 2). At this point the sweat is absorbed into the paper, which activates the battery (Step 3). Then, the electronics in the patch perform the discrimination of the power delivered by the battery (Step 4). Finally, the result of the test can be read in the electrochromic displays, the Control Display turns on to show that the test is valid, whereas the Test Display turns on only in case of a positive result (Step 5).

**Sweat conductivity as a screening parameter for Cystic Fibrosis**

Sweat conductivity has proved to be a reliable method to discriminate between CF and non-CF subjects. Conductivity measurements have proven to approximate very closely to the sum of the sodium and potassium concentrations in sweat^1^. The small bias observed between electrical conductivity and chloride concentration was found to be due to the natural presence of other species like potassium, bicarbonate, ammonium and lactate ions. This bias was observed to be function of the conductivity of the sample, and this enabled converting chloride range-limiting levels to their electrolyte conductivity equivalents shown in Table S1. Note that conductivity is expressed in mmol L^-1^, being this parameter, the conductivity that would have a solution containing the referred concentration of ions. The electrolyte typically selected as reference is sodium chloride. Therefore, conductivity values of the solutions used along the paper will be referred to mmol L^-1^ of equivalent NaCl (mᴍ NaCl).

**Table S1.** Equivalent conductivity due to the chloride content in sweat and in a solution of NaCl.

|  | **Chloride content**  **(mmol L^-1^)** | **Electrolyte concentration (mmol L^-1^)** |
| --- | --- | --- |
| **Normal** | 0-40 | 0-60 |
| **Borderline** | 40-60 | 60-80 |
| **Abnormal** | +60 | +80 |

To calibrate the battery in the most valuable range of chloride concentration for the specific application of screening cystic fibrosis the Cystic Fibrosis Foundation Guidelines for Diagnostic Sweat Testing were followed^2^. In the Guideline 17 is stated that *the lower limit of detection should be determined by the laboratory and should be ≤ 10 mmol L^-1^. The upper end of reportable results should be no more than 160 mmol L^-1^*.

**Self-powered skin-patch design**

| 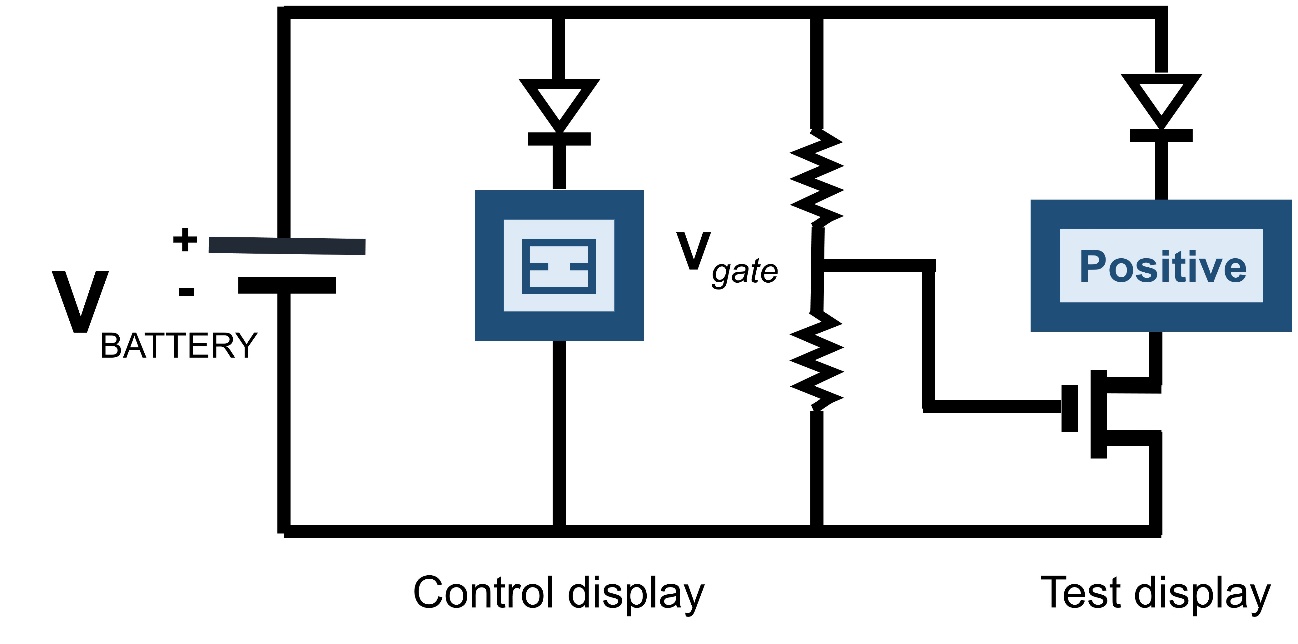 |
| --- |
| **Figure S2.** Circuit implemented in the self-powered device. |

The circuit of the self-powered patch is presented (Fig. S2). Just powered by the battery, two electrochromic displays are fed to provide an optical output to the user. Electrochromic displays were identified as the most suitable technology due to its compatibility with flexible substrates, its fabrication with screen-printing techniques and their capability to operate with voltages as low as 0.6 V. Therefore, the visualization of test results in the patch is done with two custom-made electrochromic displays (Ynvisible, Portugal); a Control display that assesses that the patch has effectively collected the sweat sample and that the battery sensor is correctly activated and a Test Display that turns on only when the sweat conductivity is equal or above the conductivity threshold level set in the application. Two diodes were placed in the circuit to avoid the display discharge and turning off. An n-type MOSFET controls the turning on of the Test Display. To match the CF-positive threshold voltage of the battery sensor with the gate Voltage (V_gate_), R_LOAD_ was divided between two resistors R_1_ and R_2_. R_2_ value was calibrated so when the voltage provided by battery was below the gate voltage, V_2_< V_gate_ and no current (i_positive_) flows towards the Test display. Contrarily, when the battery sensor yielded a voltage over the threshold, V_2_ >V_gate_, allowed the current to flow towards the Test Display turning it on.

**Transistor characterization**

The transistor used to control the switching on of the Test display is an n-type MOSFET, the transfer characteristic of which (i.e I_DS_ versus V_G_) is shown (Fig. S3). It can be seen that when applied voltages at the gate terminal are higher than 1 V the current starts flowing through the drain and source terminals of the transistor. This changes the state of the transistor from the channel off regime to the linear regime. Although there was a current already flowing at voltages as low as 1 V, the calibration of the patch showed that until the V_G_ did not achieve values over 1.2 V (and currents above 0.5 μA) the Test Display did not turn on. Therefore, 1.2 V was considered as the threshold voltage to discriminate between healthy or non-healthy samples.

| **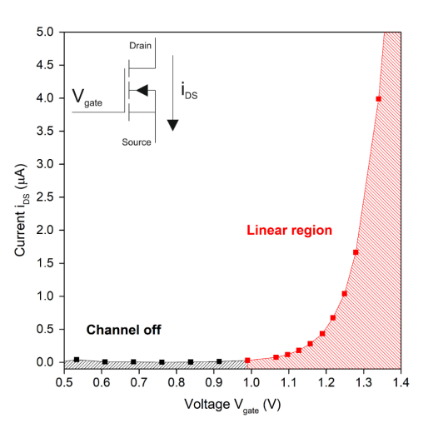** |
| --- |
| **Figure S3.** Transistor characteristic. Representation of the current flowing through the terminals of the transistor depending on the gate voltage. |

**Movie S1**. Activation of the battery upon the addition of a fluid.

**Table S2**. Composition of artificial eccrine perspiration solution.

| Compounds | Type | Quantity (g/L) |
| --- | --- | --- |
| Amino acids | Glycine | (from 0.001 for Taurine to 0.15 for Serine) |
|  | L-Alanine |  |
|  | L-Arginine |  |
|  | L-Asparagine |  |
|  | L-Aspartic acid |  |
|  | L-Citrulline |  |
|  | L-Glutamic acid |  |
|  | L-Histidine |  |
|  | L-Isoleucine |  |
|  | L-Leucine |  |
|  | L-Lysine as hydrochloride |  |
|  | L-Methionine |  |
|  | L-Ornithine as hydrochloride |  |
|  | L-Phenylalanine |  |
|  | L-Serine (Largest amount) |  |
|  | L-Threonine |  |
|  | L-Tyrosine |  |
|  | L-Valine |  |
|  | Taurine |  |
| Metabolites | Uric acid  Lactic acid | (from 0.007 for Uric Acid to 0.8 for Urea) |
|  | Urea |  |
|  | Ammonia |  |
| Minerals | Sodium | 1.80 |
|  | Calcium | 0.81 |
|  | Potassium | 2.46 |
|  | Zinc | 0.002 |
|  | Iron | 0.002 |
|  | Sulphate | 0.25 |
|  | Chloride | 2.85 |
|  | Magnesium | 0.34 |

**References**

1. Hammond, K. B., Turcios, N. L. & Gibson, L. E. Clinical evaluation of the macroduct sweat collection system and conductivity analyzer in the diagnosis of cystic fibrosis. *J. Pediatr.* **124,** 255–60 (1994).

2. LeGrys, V. A., Yankaskas, J. R., Quittell, L. M., Marshall, B. C. & Mogayzel, P. J. Diagnostic Sweat Testing: The Cystic Fibrosis Foundation Guidelines. *J. Pediatr.* **151,** 85–89 (2007).
